# Amorphous Drug–Polymer Salt with High Stability under Tropical Conditions and Fast Dissolution: The Case of Clofazimine and Poly(acrylic acid)

**DOI:** 10.1021/acs.molpharmaceut.0c01180

**Published:** 2021-02-01

**Authors:** Yue Gui, Erin C. McCann, Xin Yao, Yuhui Li, Karen J. Jones, Lian Yu

**Affiliations:** ^†^School of Pharmacy and ^‡^Zeeh Pharmaceutical Experiment Station, School of Pharmacy, University of Wisconsin−Madison, Madison, Wisconsin 53705, United States

**Keywords:** amorphous drug−polymer salt, clofazimine, poly(acrylic acid), physical stability, tropical conditions, dissolution

## Abstract

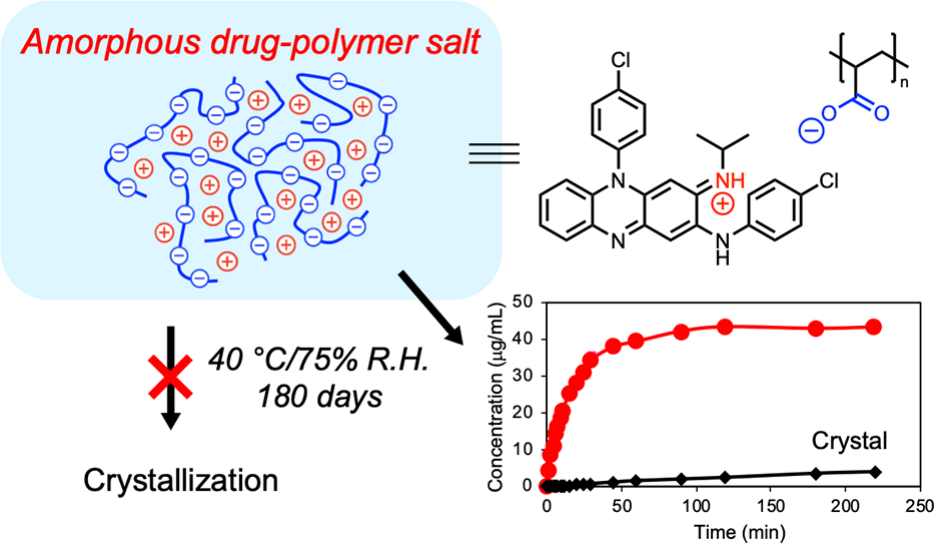

We report that the stability of amorphous clofazimine (CFZ) against crystallization is vastly improved by salt formation with a polymer without sacrificing dissolution rate. A simple slurry method was used to produce the amorphous salt of CFZ with poly(acrylic acid) (PAA) at 75 wt % drug loading. The synthesis was performed under a mild condition suitable for thermally unstable drugs and polymers. Salt formation was confirmed by visible spectroscopy and glass temperature elevation. The amorphous salt at 75 wt % drug loading is remarkably stable against crystallization at 40 °C and 75% RH for at least 180 days. In contrast, the amorphous solid dispersion containing the un-ionized CFZ dispersed in poly(vinylpyrrolidone) crystallized in 1 week under the same condition. The high stability of the amorphous drug–polymer salt is a result of the absence of a drug–polymer crystalline structure, reduced driving force for crystallizing the free base, and reduced molecular mobility. Despite the elevated stability, the amorphous drug–polymer salt showed fast dissolution and high solution concentration in two biorelevant media (SGF and FaSSIF). Additionally, the amorphous CFZ–PAA salt has improved tabletability and powder flow relative to crystalline CFZ. The CFZ–PAA example suggests a general method to prepare amorphous drugs with high physical stability under tropical conditions and fast dissolution.

## Introduction

Amorphous formulations can improve the solubility and bioavailability of poorly soluble drugs but must be stable against crystallization.^1^ Stability under the highly stressful tropical conditions is a requirement for medicines for global health. Polymers are commonly used to stabilize amorphous drugs against crystallization and to provide other benefits such as improved wetting and dissolution.^2^ While many studies employed polymers as bulk additives and dispersion media,^3,4^ there has been recent attention to using polymers as coating materials to inhibit surface crystallization and improve wetting.^5−11^ Many amorphous drugs have high surface mobility^12−14^ and show fast surface crystal growth.^15−17^ Thin polymer coatings can immobilize surface molecules, inhibit surface crystallization, and improve wetting and dissolution.

Salt formation is widely used in pharmaceutical science to improve the physical properties of drugs.^18^ Pharmaceutical salts usually contain an ionized drug with a small counterion (an inorganic ion or a small charged organic molecule). In contrast, salts formed between drugs and polymers are less well studied.^19^ For the purpose of stabilizing amorphous drugs, the formation of drug–polymer salts is expected to be advantageous for many reasons. First, ionic interactions are stronger than van der Waals forces between neutral molecules, and this can reduce the system’s free energy and the driving force for crystallization. Second, an amorphous drug–polymer salt is expected to have a much higher glass transition temperature than a neutral dispersion, again a result of strong ionic interactions. This would lead to lower molecular mobility and greater stability. Third, while many small-molecule salts can crystallize, a drug–polymer salt may be very difficult (if not impossible) to crystallize. This is because a stable crystal packing containing both the drug and the polymer may not exist. For these reasons, we expect an amorphous drug–polymer salt to be significantly more stable than the neutral drug–polymer dispersion, especially under the highly stressful tropical conditions.

There are scattered literature reports that support the notion of high amorphous stability by formation of drug–polymer salts. The basic polymer Eudragit E PO has been used to stabilize acidic drugs naproxen^20^ and indomethacin;^21^ the acidic polymer poly(acrylic acid) (PAA, Carbomer) has been used to stabilize 2-aminopyridine-containing basic drugs.^22^ The recent work on polymer nanocoating takes advantage of the *local* formation of drug–polymer salts. For example, the basic polymer chitosan is deposited on the surface of the acidic drug indomethacin,^8^ and the acidic polymer alginic acid is deposited on the surface of the basic drug clofazimine.^5^ In the coating solution, the drug and the polymer are oppositely charged, allowing salt formation. Despite these reports, the hypothesis that drug–polymer salt formation leads to high amorphous stability has not been systematically explored.

**Scheme 1:**
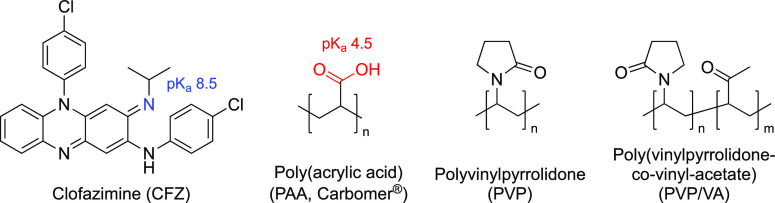
Chemical Structures of CFZ, PAA, PVP and PVP/VA The functional groups of CFZ and PAA responsible for basicity and acidity are highlighted along with p*K*_a_ values.

This work is concerned with the amorphous salt of clofazimine (CFZ) and the polymer PAA (Scheme 1). CFZ is an antimicrobial drug for treating leprosy and extensively drug-resistant tuberculosis and one of the World Health Organization’s essential medicines.^23^ CFZ is in class II of the Biopharmaceuticals Classification System (low solubility and high permeability), suggesting the potential for improved absorption by enhancing solubility. CFZ is a weak base with a p*K*_a_ of 8.5.^24^ The polymer PAA is a weak acid with a p*K*_a_ of 4.5.^25^ The large difference between their p*K*_a_ values suggests potential for salt formation.^26^

We report that the CFZ–PAA salt can be synthesized using a simple slurry method and exhibits high physical stability during storage at high temperature and humidity. The synthesis was performed under mild conditions, preventing the thermal decomposition of CFZ^27^ and PAA.^28^ Salt formation was verified by thermal analysis and spectroscopy. The amorphous salt was stable against crystallization at 40 °C and 75% RH for at least 180 days, vastly outperforming a neutral drug–polymer dispersion tested under the same condition. Despite its high stability, the amorphous drug–polymer salt showed fast dissolution and high solution concentration in two biorelevant media (simulated gastric fluid, SGF, and fasted state simulated intestinal fluid, FaSSIF) relative to crystalline CFZ.

## Materials and Methods

Clofazimine [*N*,5-bis(4-chlorophenyl)-3-(1-methylethylimino)-5*H*-phenazin-2-amine, CFZ, ≥98% pure], poly(acrylic acid) (PAA, average *M*_v_ of 450 kg/mol), polyvinylpyrrolidone (PVP K15, average *M*_w_ of 8000 g/mol), sodium dodecyl sulfate (SDS, ≥98% pure), sodium chloride, and sodium phosphate monobasic monohydrate were purchased from Sigma-Aldrich (St. Louis, MO) and used as received. Kollidon VA 64 (PVP/VA 64, average *M*_v_ of 45–70 kg/mol) was purchased from BASF.

Amorphous CFZ–PAA salt particles were prepared as follows. 2 mL of ethanol was added to the mixture of 375 mg of CFZ and 125 mg of PAA. The suspension was magnetically stirred at 75 °C maintained by a sand bath for 1 h (Fisher Thermix stirring hot plate model 301T). During reaction, the color of the solid phase in the slurry changed from red (color of CFZ crystals) to black. The solid product was filtered, washed twice with ethanol, and dried in vacuum at room temperature overnight. The product was ground in a mortar with a pestle, and particles in the size range 45–75 μm (between two sieves) were collected for characterization.

Amorphous solid dispersions of CFZ–PVP and CFZ–PVP/VA were prepared at a drug loading of 75 wt % by mixing 375 mg of CFZ and 125 mg of the dispersion polymer in an Al weighing dish and melting the mixture on a hot plate at 217–220 °C. The melt was cooled to room temperature, and the solid material was ground and sieved to obtain particles in the size range 45–75 μm.

Thin films of amorphous CFZ–PAA salt were prepared by spin coating for visible absorption spectroscopy. CFZ and PAA of known ratios were dissolved in ethanol and dichloromethane (1:1 v/v). The concentration of CFZ was 5 mg/mL. Drops of each solution were deposited on a silicate glass coverslip affixed to a spin-coater (TC100 desktop spin coater, MTI Corporation). The rotation speed was 200 rpm, and the coating time was 1 min. After coating, a transparent film was formed on the coverslip. Visible absorption spectra were collected through the films using an Agilent 8453 UV–visible spectrophotometer.

PAA-coated CFZ particles were prepared for ζ potential measurement. 100 mg of crystalline CFZ particles was placed in a 20 mL glass vial containing a magnetic stirrer and 1 mL of the PAA solution (2 mg/mL). The vial was placed on its side, and the slurry was stirred at 100 rpm for 2 min. The slurry was filtered and rinsed with the coating solution. The particles were dried in vacuum at room temperature for 3 h.

ζ potential measurements were performed with a Zetasizer Nano ZS (Malvern Instruments, USA). CFZ–PAA particles of different drug loading and PAA-coated CFZ were suspended in Milli-Q water for this measurement.

**Figure 1:**
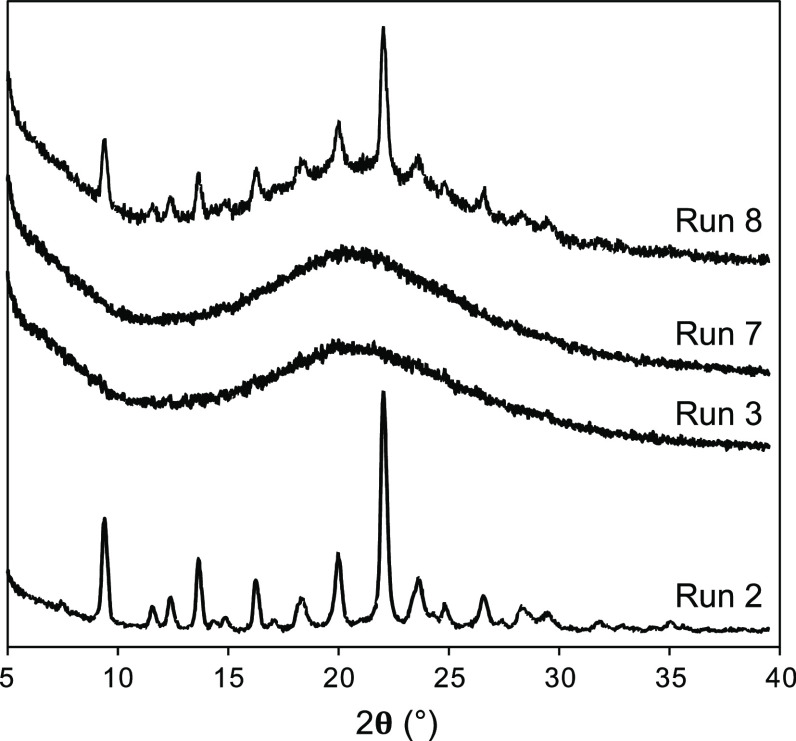
PXRD patterns of solid products from reactions performed under different conditions (see Table 2).

Crystallization of amorphous particles was monitored by powder X-ray diffraction (PXRD; Bruker D8 Advance diffractometer with a Cu Kα source, λ = 1.54178 Å; Figure 1). Single-crystal X-ray diffraction was performed with a Bruker D8 VENTURE Photon III four-circle diffractormeter with a Cu Kα source, λ = 1.54178 Å. See the deposited CIF file under the deposition number 2046715 for details of structural solution for the salt of CFZ and dodecyl sulfate (CFZ–DS), which can be obtained free of charge from the Cambridge Crystallographic Data Centre www.ccdc.cam.ac.uk/structures.

Dissolution testing was performed in two biorelevant media (Table 1), using a USP-II apparatus (paddle) at 37 °C and 100 rpm. 50 mg of CFZ–PAA particles was added in 1000 mL of SGF^29^ or 100 mL of FaSSIF (prepared according to the protocol from its powder manufacturer, Biorelevant) to represent the clinical dose.^30^ The media volume selections were based on the FDA guideline^31^ and the mean fluid volume in the small intestine at the fasted state.^32^ At each time point, 3 mL of the solution was withdrawn, filtered through a 0.2 μm syringe filter (polytetrafluoroethylene), and analyzed with a UV–visible spectrometer (Agilent 8453 UV–visible spectrophotometer) at 495 nm (SGF) or 492 nm (FaSSIF). CFZ concentration was calculated using Beer’s law against a calibration curve. After each withdrawal, 3 mL of fresh dissolution medium was added back to the dissolution vessel.

**Table 1:** Compositions in SGF and FaSSIF

SGF	FaSSIF
NaCl (43 mM)	NaCl (106 mM)
SDS (3.5 mM)	NaH_2_PO_4_ (29 mM)
HCl (0.01 N)	sodium taurocholate (3 mM)
pH 2	soybean lecithin (0.75 mM)
	NaOH (10 mM)
	pH 6.5

Differential scanning calorimetry (DSC) was conducted with a TA Instruments Q2000 at 10 °C/min under N_2_ purge (50 mL/min). Thermogravimetric analysis (TGA) was conducted at 10 °C/min in open Al pans using a TA Q600 SDT unit. ^1^H NMR was measured in *d*-DMSO using a Bruker Avance III HD 400 MHz instrument at room temperature.

To assess tabletability, approximately 50 mg of powder was filled into a 6 mm diameter die and compressed using flat-faced punches on a Carver Press Auto M3890. Tablets were allowed to relax for 24 h under ambient conditions before testing. The diametric breaking force was measured using a Benchsaver series VK 200 tablet hardness tester. Tablet tensile strength σ (MPa) was calculated from the maximum breaking force *F* (N), tablet diameter *D* (m), and tablet thickness *T* (m) as follows:^33^
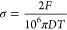
Angle of repose was measured by pouring 500 mg of powder through a funnel whose outlet was 4 mm inside diameter and placed 1 in. above a horizontal receiving surface. A picture was taken of the rested powder from the side, and the angle of repose was measured from the image.

## Results and Discussion

### Synthesis

In our synthesis of the amorphous CFZ–PAA salt, CFZ crystals reacted with PAA in a slurry to produce an amorphous solid. During the reaction, the initially red crystals of CFZ turned black. We used the product’s degree of crystallinity, measured by PXRD, as a measure to optimize reaction conditions, with a goal of obtaining fully amorphous product in a short time at a high drug loading. The parameters to be optimized included reaction temperature and solvent.

In Table 2, runs 1–4 were all conducted at 50% drug loading at 50 °C for 60 min but with different solvents. With water as solvent (or without solvent), no reaction was observed (no loss of CFZ crystallinity; see runs 1 and 2). With ethanol or acetone as solvent, crystalline CFZ completely turned amorphous (runs 3 and 4). This is attributed to the fact that ethanol and acetone are better solvents of CFZ than water. We chose ethanol as the solvent for further development given its lower toxicity and environmental impact.^34^

**Table 2:** Experiments to Optimize Synthetic Conditions

run	drug loading (wt %)	solvent	temperature (°C)	time (min)	% crystallinity
1	50	none	50	60	100
2	50	water	50	60	100
3	50	ethanol	50	60	0
4	50	acetone	50	60	0
5	50	ethanol	23	1440	80
6	50	ethanol	75	1	0
7	75	ethanol	75	60	0
8	80	ethanol	75	60	25

Runs 3, 5, and 6 were used to optimize reaction temperature. These runs were all performed at 50 wt % drug loading and with ethanol as solvent. Reaction was complete in 1 min at 75 °C (run 6) and in 60 min at 50 °C (run 3) but was 20% complete after 24 h at 23 °C (run 5). 75 °C was chosen as the temperature for synthesis.

Under the chosen reaction conditions, 75 wt % CFZ (run 7) was the maximal drug loading obtainable. A further increase to 80 wt % resulted in unreacted crystals (run 8). 75 wt % drug loading corresponds to a molar ratio 1:2 for CFZ:PAA monomer (the molecular weight of clofazimine is 473 g/mol, and that of the monomer of PAA is 72 g/mol). This high drug loading exceeds those reported previously for amorphous drug-polymer salts.^20−22^

No chemical degradation of the drug occurred during synthesis. This was demonstrated by analysis by ^1^H NMR (Figure S1 in Supporting Information). This is not surprising given the reaction temperature 75 °C is well below the melting point of CFZ, 221 °C, near which the drug does decompose rapidly. The mild conditions employed in our method are suitable for thermally unstable drugs and polymers and can be easily deployed in developing countries.

### Salt Formation

**Figure 2:**
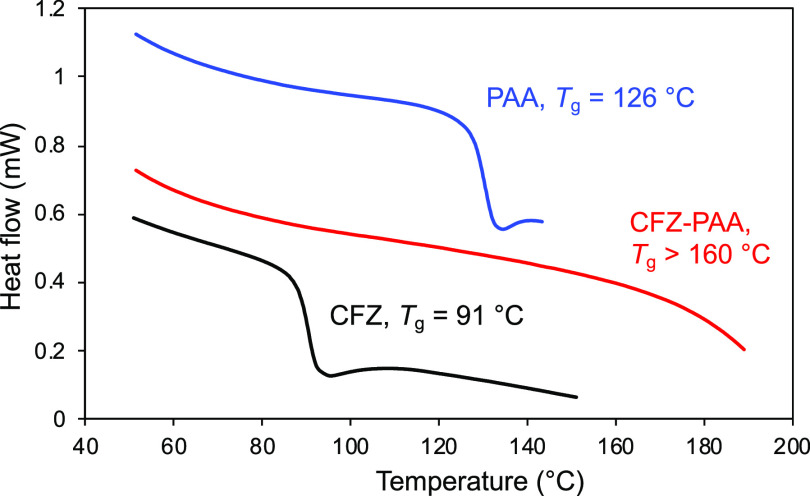
Glass transitions in PAA, amorphous CFZ–PAA salt, and CFZ detected by DSC.

Salt formation between CFZ and PAA is indicated by *T*_g_ elevation and visible spectroscopy. Figure 2 shows the DSC result of amorphous CFZ–PAA particles along with the results of CFZ and PAA. Glass transitions are detected in CFZ and PAA as steps in heat flow at 91 and 126 °C, respectively. In contrast, no transition is detected in CFZ–PAA in the same temperature range. These data indicate the glass transition temperature (*T*_g_) of CFZ–PAA must be above the *T*_g_ values of the components; it must be higher than 160 °C, above which CFZ and PAA decompose, obscuring detection. For a drug–polymer dispersion without salt formation, *T*_g_ usually falls between the component *T*_g_ values, conforming to mixing rules like the Fox equation. The elevated *T*_g_ relative to the pure components indicates strong interactions between CFZ and PAA, consistent with ionic interactions and salt formation.^35^

**Figure 3:**
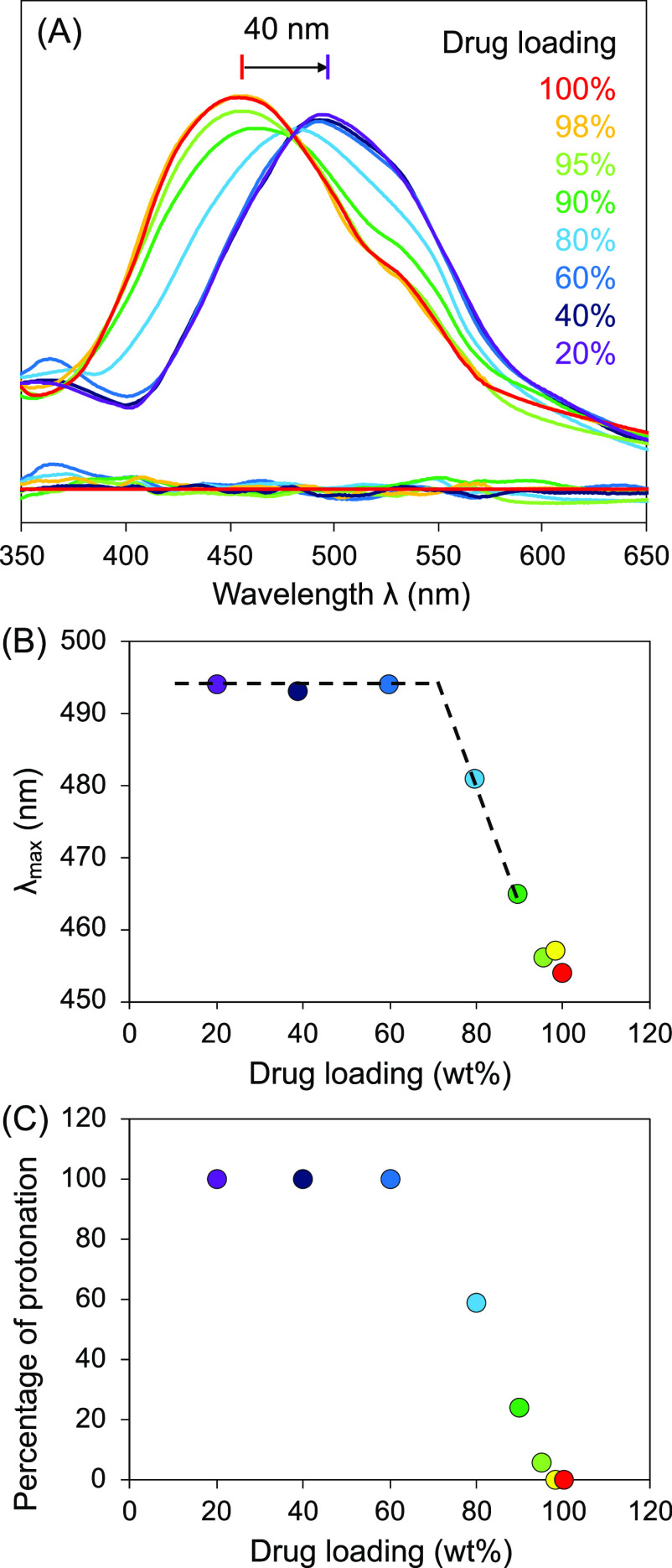
(A) Visible absorption spectra of amorphous CFZ–PAA films at different drug concentration. (B) λ_max_ (wavelength of maximal absorption) vs drug concentration. The color of each data point corresponds to the spectrum in (A) of the same color. By extrapolation, the saturation drug concentration is determined at 70 wt %. (C) Percentage of protonated CFZ vs drug loading by fitting the spectra in (A) to a two-state model.

Figure 3A shows the visible absorption spectra of amorphous CFZ–PAA films at different concentrations. Pure CFZ has the strongest absorption at λ_max_ = 452 nm. With addition of PAA, the absorption peak shifts to a longer wavelength; the shift increases and saturates at 493 nm as drug concentration is reduced below 60 wt %. This saturation behavior is shown in Figure 3B where λ_max_ is plotted against drug concentration. By extrapolation, the drug concentration at which saturation occurs is 70 wt %. This spectral shift results in a change of film color: pure CFZ is red, and the addition of PAA deepens the color, eventually making it dark purple. Similar spectral changes have been reported for CFZ in the presence of the polymer HPMCP, which also has carboxylic acid groups able to form a salt with CFZ.^26^ A noteworthy feature in Figure 3A is the isosbestic point: despite their differences, all the spectra intersect at 480 nm.

All these results are indicative of salt formation. An acid–base reaction between PAA and CFZ means that at each concentration, the drug can exist as the unreacted free base and as the protonated conjugate acid. These two species have different spectra, and the spectrum at each concentration can be represented as the weighted average of the spectra of the free base and the conjugated acid. This two-state model can fit all the observed spectra; see the residuals of fitting at the bottom of Figure 3A, which are small relative to the spectral intensity. The two-state model also accounts for the isosbestic point in Figure 3A: this is the crossing point of the spectra of the protonated and unprotonated CFZ. From the two-state model fitting, we obtain the percentage of CFZ that is protonated at each drug loading (Figure 3C). Pure CFZ is unprotonated; with the addition of PAA (decreasing drug loading), the fraction of protonation increases; protonation is complete below 70 wt % drug loading. This saturation behavior arises from the stoichiometry of the salt. At high drug concentration, there is excess free base; at low drug concentration, all the free base has reacted with PAA and the only spectrum observed is that of the salt. As a result, the spectrum shifts with increasing concentration of PAA but the effect saturates at high enough PAA concentration. It is worth noting that the saturation limit for λ_max_, 70 wt % drug, is close to the synthetic limit, 75 wt %, for drug loading in amorphous CFZ–PAA salts.

The red-shift of the absorption spectrum of CFZ is also consistent with salt formation. The absorption of CFZ at λ_max_ = 452 nm is an excitation of the π electron system. Protonation at the imine site (Scheme 1) introduces a positive charge, pulling π electrons toward the charge. This leads to a change in electronic energy levels and a red-shift of the spectrum.^36^

Taken together, the elevation of *T*_g_ and the spectral change both indicate salt formation between CFZ and PAA. This conclusion is consistent with the large difference between the p*K*_a_ values of the two components: the base CFZ has a p*K*_a_ of 8.5; the acid PAA has a p*K*_a_ of 4.5; they are expected to form a salt according to the rule^37,38^ that proton transfer can happen when the p*K*_a_ difference exceeds 2.

### Stability at High Temperature and Humidity against Crystallization

**Figure 4:**
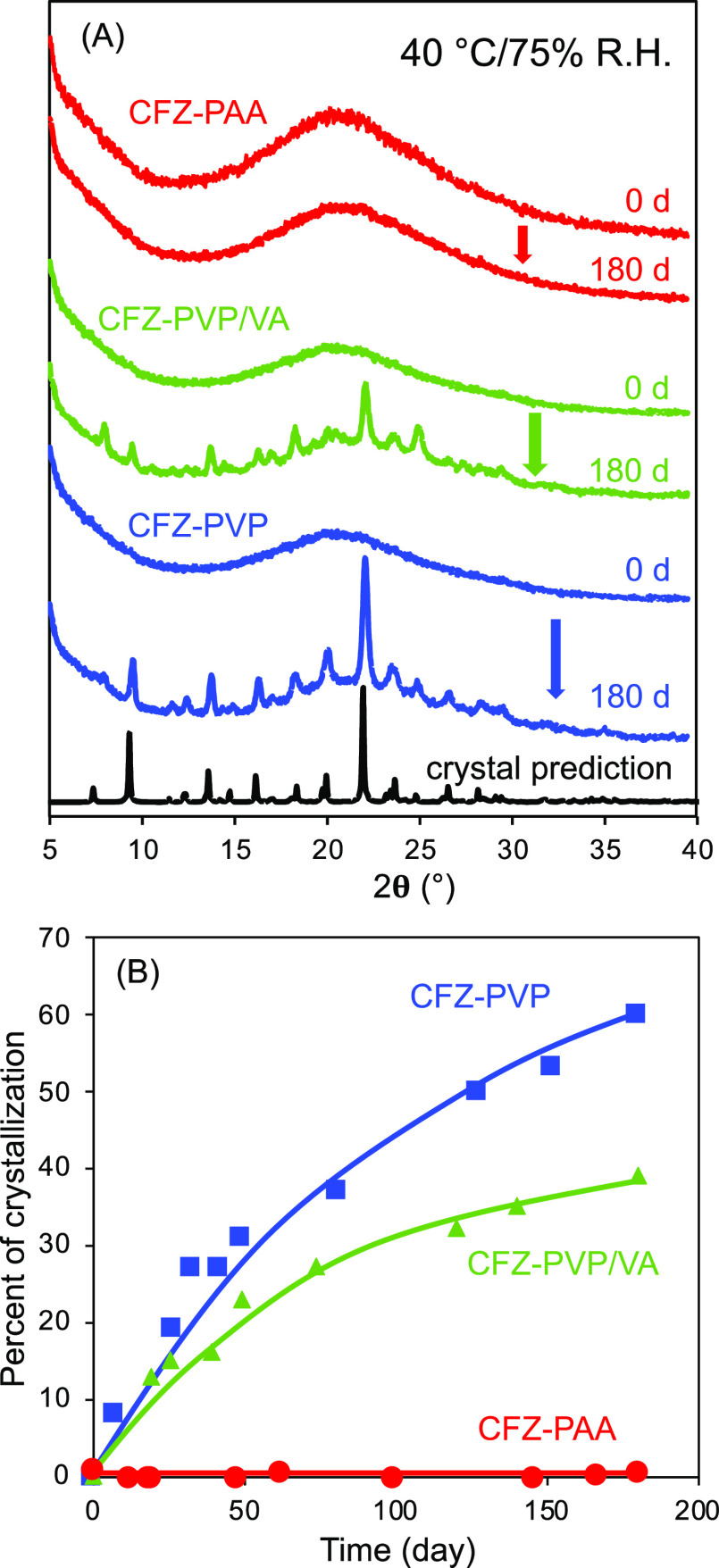
Physical stability of amorphous CFZ–PAA salt at 40 °C and 75% RH. (A) PXRD patterns before and after storage of the CFZ–PAA salt and of the neutral CFZ–PVP and CFZ–PVP/VA dispersions. The CFZ–PAA salt remained amorphous after 180 days, but the neutral CFZ–PVP and CFZ–PVP/VA dispersions crystallized as the free base (bottom trace). (B) Crystallinity change as a function of time.

The amorphous CFZ–PAA salt has remarkable stability against crystallization during storage at high temperature and humidity. Figure 4 shows that at 75 wt % drug loading, the salt remains amorphous after 180 days at 40 °C and 75% RH. This passes the accelerated stability testing for all climate zones.^39^ In contrast, at the same drug loading, the neutral CFZ–PVP and CFZ–PVP/VA dispersions show significant crystallization under the same condition. PVP and PVP/VA are commonly used dispersion polymers and serve as a reference for PAA. Figure 4B shows the change of crystallinity as a function of time. While the CFZ–PAA salt shows no crystallization, the neutral CFZ–PVP and CFZ–PVP/VA dispersions are 60% and 40% crystallized, respectively.

It is noteworthy that the amorphous CFZ–PAA salt is stable against crystallization even after absorbing a significant amount of water. The high humidity in tropical climate causes drug products to absorb moisture. During storage at 40 °C and 75% RH, the water content in the CFZ–PAA salt increases to 5 wt % from the initial 1 wt % (Figure S2). Despite this, the amorphous salt remains stable against crystallization.

### Dissolution Rate

**Figure 5:**
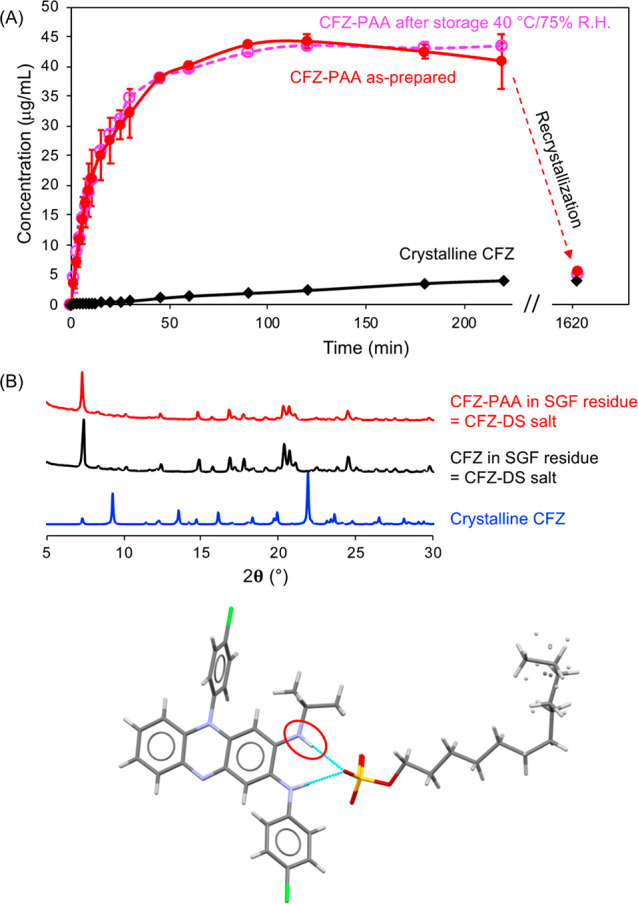
(A) Dissolution kinetics of crystalline CFZ and amorphous CFZ–PAA salt as prepared and after 180 days at 40 °C and 75% RH in SGF. 75 wt % drug loading in the amorphous salt. The error bar indicates standard deviation of two independent preparations tested. (B) PXRD patterns of solid residues after dissolution in SGF. The crystalline CFZ and the amorphous CFZ–PAA salt both transformed to a crystalline CFZ–DS salt.

The amorphous CFZ–PAA salt shows fast dissolution in two biorelevant media, SGF and FaSSIF. In SGF, amorphous CFZ–PAA salt dissolves much faster than the crystalline CFZ of the same particle size tested under the same condition (Figure 5). After 2 h, the salt reaches a solution concentration 20 times higher than that reached by crystalline CFZ. We interpret the plateau concentration, 45 μg/mL, as the solubility of the amorphous salt in SGF. This solubility is 10 times higher than the solubility of crystalline CFZ, 4 μg/mL.^5^ The high solution concentration is sustained for at least 3 h, resulting in an enhancement by a factor of 20 of the area under the curve within the gastric emptying time (4 h).^40^ It should be emphasized that the enhanced dissolution rate is unaffected by storage at 40 °C and 75% RH (see the pink curve in Figure 5A). This is another evidence for the high stability of the drug–polymer amorphous salt under the highly stressful conditions of 40 °C and 75% RH.

Upon prolonged contact with SGF and stirring, the amorphous CFZ–PAA salt gradually crystallized, leading to reduced solution concentration. After 27 h, the concentration was reduced to 4.2 μg/mL, the same concentration reached by crystalline CFZ. In both cases, analysis of the solid residues indicated a crystalline material different from the CFZ free base (Figure 5B). This solid material proved to be a salt of CFZ with dodecyl sulfate (DS, a component of SGF). In the crystal structure, the drug is protonated (see the circled site in the molecular structure), consistent with the ability of CFZ to form salts (see the CIF in the Supporting Information for details). An intriguing observation is that the CFZ–DS salt crystals are thin and easily bent and twisted (Figure S3), a phenomenon of some recent interest.^41^

**Figure 6:**
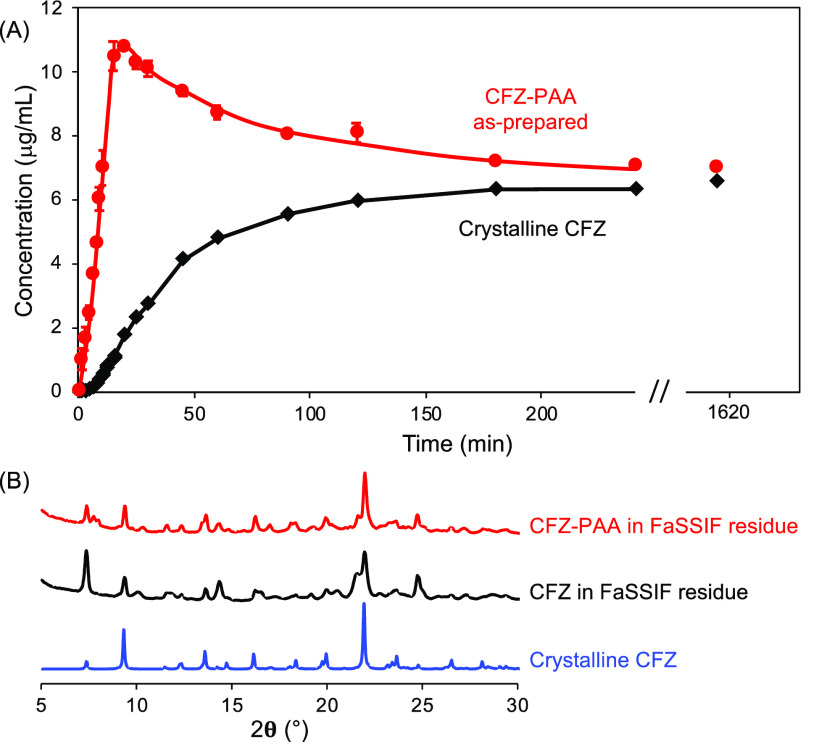
(A) Dissolution kinetics of amorphous CFZ–PAA salt and crystalline CFZ in FaSSIF. 75 wt % drug loading in the amorphous salt. (B) PXRD of solid residues after dissolution in FaSSIF. They are mostly crystalline CFZ.

The amorphous CFZ–PAA also shows fast dissolution in FaSSIF relative to crystalline CFZ (Figure 6A). At 75 wt % drug loading, the amorphous salt dissolves 10 times faster than crystalline CFZ in the first 15 min. The amorphous salt reaches a maximal solution concentration at 20 min, after which the concentration decreases and approaches the solubility of crystalline CFZ. The area under the curve for amorphous CFZ–PAA is 1.5 times that for crystalline CFZ within the small intestinal transit time (4 h).^42^ Analysis of the solid residues after dissolution indicated mostly crystalline CFZ (Figure 6B). This common solid form determined the final drug concentration in FaSSIF. An interesting difference between the dissolution kinetics in the two media used is that the onset of crystallization is sooner in FaSSIF than in SGF. This may reflect the different nucleation rates of the CFZ free base and the CFZ–DS salt.

### Does PAA Form a Surface Coating?

A polyelectrolyte similar to PAA, alginic acid, has been used in a surface coating on CFZ^5^ and other amorphous drugs by electrostatic deposition.^8^ The coating process involves dipping CFZ in a polymer solution at a pH such that the two components are oppositely charged. Given the coating conditions are similar to the conditions of synthesizing the amorphous salt, it is of interest to determine whether the amorphous salt contains a surface coating of PAA. We answer this question by measuring the ζ potential of the amorphous salt. Previous work has shown that amorphous CFZ can be coated by alginic acid and the coating changes the ζ potential in water from +44 mV for CFZ to −50 mV for alginic-acid-coated CFZ. If the amorphous salt has a surface coating of PAA, a negative ζ potential should be observed at high drug loading.

**Figure 7:**
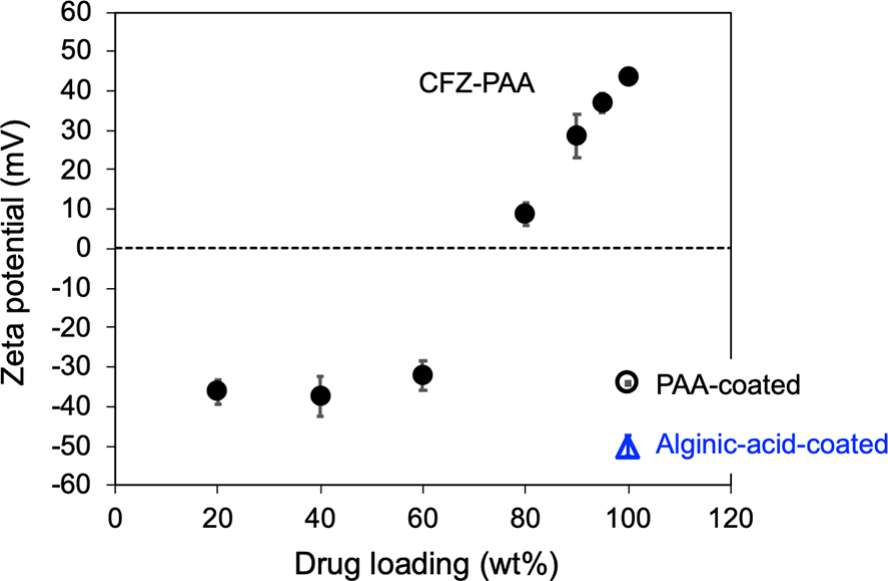
ζ potentials of the amorphous CFZ–PAA salt as a function of drug loading and of CFZ particles coated with PAA and alginic acid, as labeled. Error bar is the standard deviation for three measurements.

Figure 7 shows the ζ potential of the amorphous CFZ–PAA salt particles dispersed in pure water. Pure CFZ particles have a positive surface charge (+44 mV). This is expected for a basic drug with a p*K*_a_ of 8.5 at neutral pH; the drug is protonated, gaining a positive charge. With the addition of PAA, the surface potential decreases, eventually becoming negative near 70 wt %. Below 60 wt % drug concentration, the surface potential equilibrates near −37 mV. This is a result of the neutralization of the positive charge of CFZ by the negative charge of PAA and by the dilution of CFZ by PAA. PAA is an acid with a p*K*_a_ of 4.5 and is negatively charged at neutral pH. By salt formation, PAA neutralizes the charges of CFZ molecules. With enough PAA added, all CFZ charges are neutralized and the surface charge is dictated by the charge of PAA, which is negative.^43^

Figure 7 also shows the ζ potentials of CFZ particles coated by PAA and alginic acid. With a polymer surface coating, the ζ potential of CFZ becomes negative, as expected. Since the polymer coating is only several nanometers thin, these data points are placed in the figure near 100% drug loading, as the coated particles are almost pure CFZ. The very thin surface coating is consistent with the observation that there is no significant change of the red color of CFZ particles after coating, whereas upon salt formation in the bulk, the particle color changes from red to black.

An important conclusion we draw from Figure 7 is that the amorphous CFZ–PAA salt particles have no surface coating of PAA. The state of surface charge closely tracks the state of ionization in the bulk (Figure 3). From Figure 3, we saw that in the bulk, complete neutralization of the drug occurs at 70% drug concentration. This is the same concentration at which the surface charge changes sign (Figure 7). This argues that there is no strong surface enrichment or depletion effect for the polymer in the amorphous salt. Together, the results on surface coating and bulk doping indicate many possibilities to incorporate a polymer into an amorphous drug, so it is mostly on the surface or in the bulk. The ability to manipulate the polymer’s location in this way provides flexibility to engineer amorphous formulations. This ability is related to the low mobility of polymer chains. An interesting question for future work is, what is the equilibrium location for trace polymer in an amorphous drug?

### Tabletability and Powder Flow

**Figure 8:**
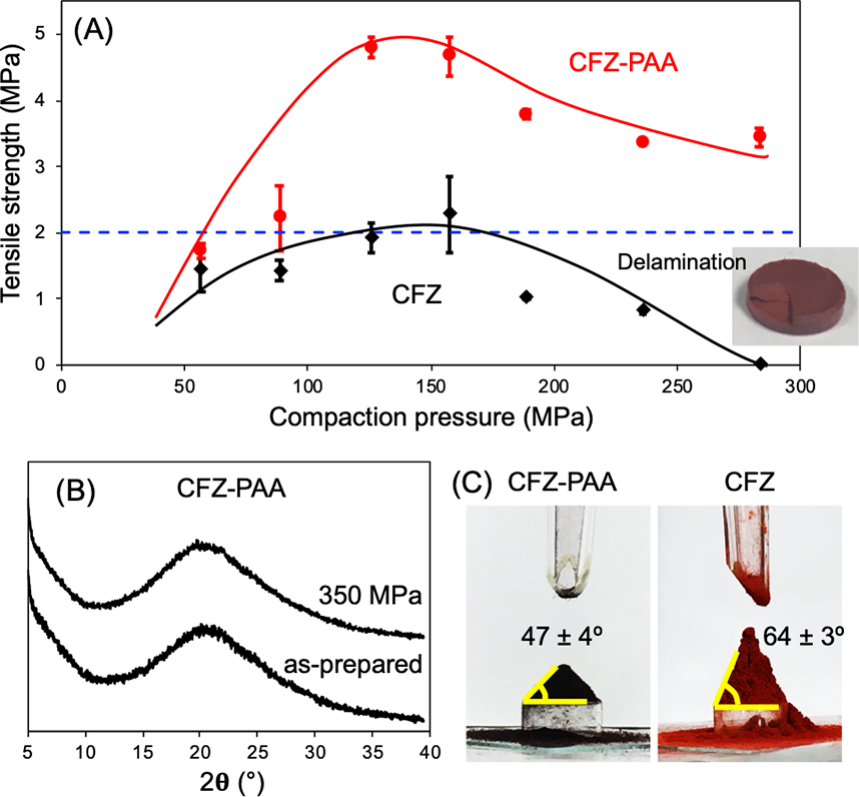
(A) Tensile strength of tablets prepared with amorphous CFZ–PAA (75 wt % drug loading) and crystalline CFZ. The amorphous salt produces stronger tablets at a given compaction pressure. (B) PXRD patterns of amorphous CFZ–PAA salt before and after compaction, indicating no crystallization during compaction. (C) Angles of repose of amorphous CFZ–PAA salt (75 wt % drug loading) and the physical mixture of CFZ and PAA.

The amorphous CFZ–PAA salt shows improved tabletability and powder flow relative to crystalline CFZ. Figure 8A shows the tablet tensile strength as a function of compaction pressure. Amorphous CFZ–PAA salt produces stronger tablets than crystalline CFZ when compared at the same compaction pressure. The strongest CFZ tablet, prepared at 150 MPa, barely meets the acceptable tensile strength of 2 MPa, while the tablet prepared with the amorphous salt is twice strong. This improvement of tabletability is likely a result of the better tabletability of the polymer.^44^ We observed no compaction-induced crystallization of the amorphous salt, even at a compaction pressure outside the normal range (350 MPa; see Figure 8B).

Figure 8C compares the angles of repose of the amorphous CFZ–PAA salt and the physical mixture of crystalline CFZ and PAA at the same drug loading (75 wt %). The amorphous salt has a smaller angle of repose than the physical mixture, indicating improved flowability upon salt formation. Good flowability is important for producing tablets and capsules at high speed with content uniformity.^45^

## Discussion

A key finding of this work is the high stability of the amorphous CFZ–PAA salt at high temperature and humidity. The salt remained amorphous after 180 days at 40 °C and 75% RH, whereas the neutral CFZ–PVP and CFZ–PVP/VA dispersions crystallized significantly under the same condition. This high stability was observed despite the significant uptake of moisture during storage. Our finding is consistent with scattered literature reports for stability enhancement by complexation between acidic drugs and basic polymers^20,21^ or between a zwitterionic drug and an acidic polymer,^46^ but in this study, drug loading was significantly higher and stability testing was performed for the longest time at 40 °C and 75% RH. These results suggest that the use of drug–polymer salts can vastly improve the stability of amorphous drugs against crystallization. We now discuss why amorphous drug–polymer salts provide high stability in this regard.

**Figure 9:**
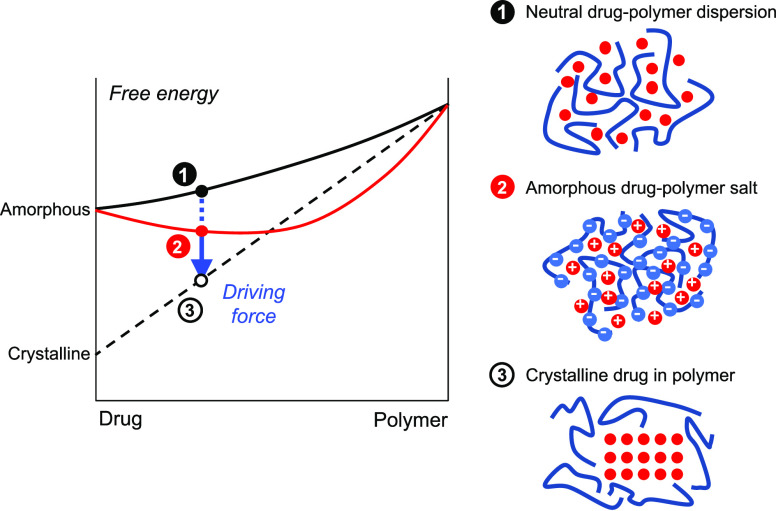
Enhanced stability of amorphous drug–polymer salts against crystallization. The curves represent the free energies of mixing to form (1) a neutral drug dispersed in a neutral polymer, (2) an amorphous drug–polymer salt, and (3) neutral-drug crystals in a polymer matrix.

We attribute the high stability of amorphous drug–polymer salts against crystallization to (1) reduced thermodynamic driving force and (2) increased kinetic barrier. Figure 9 shows the free energy of mixing in a drug–polymer system. Curve 1 represents the mixing of a neutral drug and a neutral polymer (e.g., CFZ in PVP). Curve 2 represents the mixing of a drug and a polymer where mutual ionization (salt formation) occurs; in the case illustrated, a basic drug is protonated by an acidic polymer. Curve 3 represents a mixture of the crystalline free base in a polymer matrix. The drawings to the right illustrate the three structures. In principle, a fourth structure is possible in which the drug–polymer salt crystallizes, but this is unlikely given the difficulty for the ionized drug and the ionized polymer to pack in regular arrays to form a crystal. That is, the only viable pathway of crystallization is the formation of a neutral-drug crystalline phase embedded in a polymer matrix (structure 3). Because of the strong ionic interactions in a drug–polymer salt, curve 2 is expected to be below curve 1. This means that the driving force for crystallization (arrow toward curve 3) is reduced or even nonexistent. This is the thermodynamic reason for the strong resistance of an amorphous drug–polymer salt to crystallization.

From a kinetic standpoint, salt formation elevates the glass transition temperature *T*_g_ of the drug–polymer mixture to a greater extent than simply mixing the components. In the case of CFZ, *T*_g_ is 86 °C for a neutral dispersion in PVP at 75% drug loading but is above 160 °C upon salt formation at the same drug loading. This elevation of *T*_g_ means reduced mobility and enhanced kinetic barrier for crystallization. This provides the kinetic reason for the strong resistance of an amorphous drug–polymer salt to crystallization. Together, thermodynamics and kinetics combine to make the CFZ–PAA salt exceptionally stable against crystallization at high temperature and humidity. It is likely that this principle applies in general to other drug–polymer salts.

## Conclusions

The amorphous salt of the basic drug CFZ and the acidic polymer PAA can be synthesized using a simple slurry method under mild conditions. This method is easy to implement and suitable for thermally unstable drugs and polymers. Salt formation is indicated by visible spectroscopy and *T*_g_ elevation. The amorphous drug–polymer salt is remarkably stable against crystallization under the highly stressful conditions of 40 °C and 75% RH. The high drug loading achieved exceeds the levels reported previously.^20−22^ Despite elevated stability, the amorphous salt shows fast dissolution in biorelevant media. Furthermore, the amorphous CFZ–PAA salt shows improved tabletability and powder flow relative to crystalline CFZ.

We attribute the high stability of the amorphous CFZ–PAA salt under harshly stressful conditions to reduced thermodynamic driving force and increased kinetic stability. The strong ionic interaction in a drug–polymer salt makes the free energy of mixing more negative relative to a neutral drug–polymer dispersion. This in turn reduces the driving force for crystallization. From a kinetic standpoint, salt formation elevates the glass transition temperature to a greater extent than dispersing a neutral drug in a polymer matrix. This reduces molecular mobility and enhances kinetic stability. Given the generality of these effects, we expect salt formation to provide a general approach to stabilizing amorphous drugs against crystallization, especially under the highly stressful tropical conditions for global health applications.

## Supporting Information Available

^1^H NMR of amorphous CFZ–PAA salt; TGA of amorphous CFZ–PAA salt; structure of CFZ–DS crystal (PDF)
Crystallographic data for CFZ–DS at 100 K (CIF)

## Supplementary Material

mp0c01180_si_001.pdf

mp0c01180_si_002.cif

## Abbreviations

CFZ: clofazimine
PAA: poly(acrylic acid)
PVP: polyvinylpyrrolidone
PVP/VA: poly(vinylpyrrolidone-*co*-vinyl-acetate)
SDS: sodium dodecyl sulfate
SGF: simulated gastric fluid
FaSSIF: fasted state simulated intestinal fluid; *T*_g_, glass transition temperature
