# Supplementary material for: Amorphous Drug–Polymer Salt with High Stability under Tropical Conditions and Fast Dissolution: The Case of Clofazimine and Poly(acrylic acid)
Source: Mol Pharm. 2021 Feb 1;18(3):1364–72. doi: 10.1021/acs.molpharmaceut.0c01180 (PMC7927142; doi:10.1021/acs.molpharmaceut.0c01180)
Supplement: Supplementary file 1 — mp0c01180_si_001.pdf [file mp0c01180_si_001.pdf]

Amorphous drug-polymer salt with high stability under tropical conditions and fast dissolution: The case of clofazimine and poly(acrylic acid)

Yue Gui,[1] Erin C. McCann,[1] Xin Yao,[1] Yuhui Li,[1] Karen J. Jones,[2] Lian Yu*[1]

[1] School of Pharmacy, [2] Zeeh Pharmaceutical Experiment Station, School of Pharmacy, University of Wisconsin-Madison, Madison, WI 53705, USA

Supporting Information

I. ^{1}H-NMR of amorphous CFZ-PAA salt ………………………………………………….... S1

II. TGA of amorphous CFZ-PAA salt ………………………………………………………… S2

III. Structure of CFZ-DS crystal ………………………………………………………………. S2

I. ^{1}H-NMR of amorphous CFZ-PAA salt

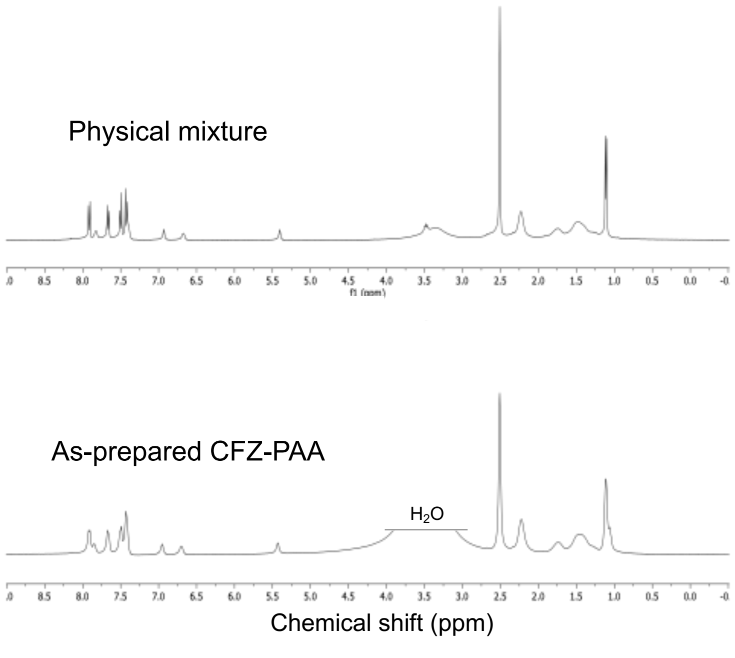

Figure S1. ^{1}H-NMR shows no decomposition after preparation of amorphous CFZ-PAA (75 wt % drug) salt.

II. TGA of amorphous CFZ-PAA salt

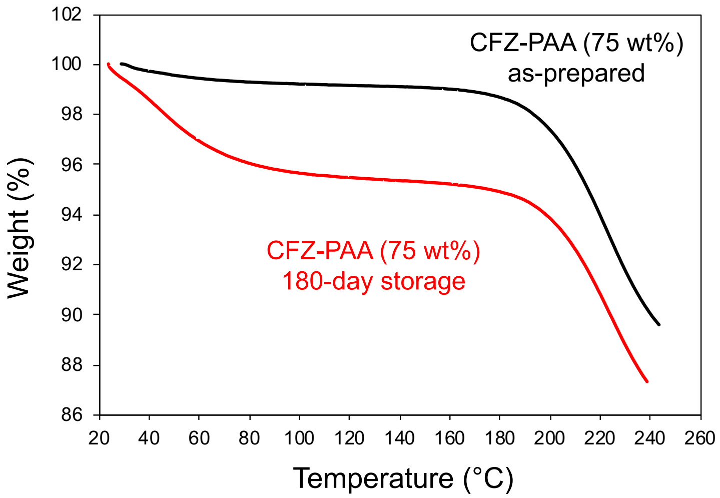

Figure S2. Water content of amorphous CFZ-PAA salt before (black) and after (red) tropical conditions (40 °C and 75% RH) storage detected by TGA. Comparing the weight loss at 100 °C, water content in amorphous CFZ-PAA salt increases from 1 wt % to 5 wt % after 180 d storage.

III. Structure of CFZ-DS crystal

Table S1. Crystal structure of CFZ-DS salt.

Crystal	CFZ-DS salt
T, K	100
space group	$P\bar{1}$
a, Å	10.924
b, Å	13.785
c, Å	14.622
α, deg	116.39
β, deg	91.27
γ, deg	108.04
V, Å^3	1842.24
Z/*Z*'	2/1
R%	5.3

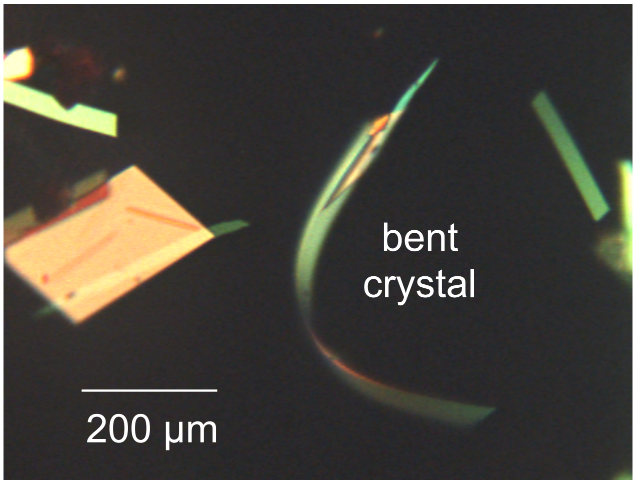

Figure S3. CFZ-DS crystals suspended in SGF are flexible and can bend in the media. The picture was taken using a polarized light microscope for an SGF droplet containing CFZ-DS crystals.
